# A meta-analysis of weight gain in first year university students: is freshman 15 a myth?

**DOI:** 10.1186/s40608-015-0051-7

**Published:** 2015-05-28

**Authors:** Claudia Vadeboncoeur, Nicholas Townsend, Charlie Foster

**Affiliations:** Nuffield Department of Population Health, University of Oxford, Oxford, UK

## Abstract

**Background:**

Observational studies report that as students transfer from secondary school to university, there is a tendency to gain weight. This phenomenon is known as the “Freshman 15” in North America, referring to the claim that on average weight gain is 15 lb (6.8 kg) in the first year of university. Studies since 1985 have mostly found weight gains ranging from 1 kg to 6 kg. Our meta-analysis aimed to update the literature on the “Freshman 15” in the first year of university. We also aimed to explore weight gain in only those who gained weight and perform several subgroup analyses. Given adolescent weight gain is highly linked to overweight and obesity in adults, a better understanding of university student weight gain is crucial if we are to combat the rising adult obesity prevalence.

**Methods:**

We conducted a search on six standard electronic databases (including PubMed, Embase, PsycInfo) from 1980 to 2014. Only peer reviewed articles with data from longitudinal studies were included. Screening was performed by two reviewers. The quality of papers was assessed and data extraction was done with a systematic approach.

**Results:**

Thirty two studies were included and 22 studies (5549 students) were included in a pooled mean meta-analysis as they reported standard errors. The overall pooled mean weight gain was 1.36 kg (3lbs) (95 % CI: 1.15 – 1.57) over an average of 5 months. A majority of students, 60.9 %, gained weight during freshman year and these on average gained 3.38 kg (7.5lbs) (95 % CI: 2.85 – 3.92).

**Conclusion:**

Freshman weight gain is an issue with almost two thirds of students gaining weight. Students who gained weight, gained it at rates much faster than in the general population. Despite most universities having some health promotion policies, we denote a consistent weight gain in university students across several countries.

## Background

As obesity has reached the level of epidemic proportions according to the World Health Organization, with an approximate number of 1.4 billion worldwide overweight and 300 million persons clinically obese [1], governments and health organizations are trying to combine efforts to curb this uprising risk factor for cardiac, endocrine and cancer diseases [2]. Adolescent obesity in particular has been shown to be a growing problem. For example, in the United States, obesity in 12–19 years old has increased dramatically in the past decades with about 35 % being overweight or obese in 2011 [3]. In those late adolescent years and early adulthood, transition from secondary school to university is a critical and vulnerable period for body weight changes and unhealthy lifestyle adoption [4–6]. Indeed, it a significant life altering moment with students being under high academic pressure, all the while having unprecedented freedom and often living away from home. In the past decade, lifestyle changes and possible predictors of weight changes during the transition have been studied. Two reviews on the topic have made a compilation of evaluated predictors of weight gain in first year students [4, 5]. Stress, alcohol drinking, unhealthy eating and physical activity decline are thought to play key roles.

The phenomenon of weight gain in the first year of university has often been referred as “Freshman 15”. This is in reference to the claim that on average, students reported gaining 15 lb (6.8 kg) in their first year of university [7]. Research conducted since 1985 have actually found weight changes ranging from−0.68 kg to 4 kg [6, 8–55] with often the first term being the most critical period. A meta-analysis on the topic from 2009 found a mean weight gain of 1.75 kg [4], far from the reported 6.8 kg (15lbs). Studies were also mixed on whether there was a gender effect, with some revealing an effect [36] but others none [18,28]. Nevertheless, this almost 2 kg weight gain over eight months is statistically significant and is higher than the weight gain rate in the general population [16, 27]. Given adolescence weight gain is highly linked to overweight and obesity in adults [56], the significant weight gain at university needs to be further understood if we are to combat the rising adult obesity prevalence. Indeed, weight gain occurring during that critical period may persist and poor life habits may settle in for the adulthood.

Our study aimed to update the literature on weight gain during the first months and first year of university of first year university students and to provide a precise and high quality estimate of this weight gain as new studies have recently been published, most with large sample sizes. Our meta-analysis included only prospective studies and further aimed to examine effects within different subgroups and more specifically, for the first time, investigated weight gain averages in weight gainers only. A better understanding of the tendency of students to gain weight in their first months at university and more specifically in those who gain weight, can help develop health policies to change the current trends. We also tested the hypothesis of gender effect and that the first term was the most critical for weight gain.

## Methods

### Search strategy and study selection

The protocol for the systematic review was published on Prospero [57]. We conducted a systematic search in six databases (PubMed, Embase, PsycInfo, CINHAL, LILCAS, Web of Science) to retrieve relevant peer-reviewed publications of empirical studies. Databases were searched from the earliest record to the search date, January 1st, 2014. The search terms included three headings: (1) Student (or Fresher* or Freshman or Freshmen), (2) Universities (or universit* or college or “higher education”) and (3) Weight (or “weight gain*” or “weight change*”, “weight increase*” or BMI or “body mass index”). Abstracts were exported to EndNote X7.

### Eligibility criteria

The main author screened titles and abstracts and a second author independently screened a random subset of 10 % of the titles and abstracts and 5 % of those identified as ‘Not Relevant’ by the primary author. Discrepancies were discussed with the third author. To be eligible for full review, studies had to be prospective and longitudinal, be conducted on first year undergraduate university students and collect weight data at baseline and follow-up. We also scanned the references of studies subject to full review for potential studies to be included. We had no language restriction. We excluded articles if (1) the population was not representative of a general first year student population (ex: a military university), if (2) follow-up was shorter than 4 weeks or longer than 8 months, if (3) a complete case analysis was not performed, if (4) the initial data collection was not performed at the beginning of the term, if (5) no weight change data was reported as a variable (6) different data collection methods were used at baseline and follow-up.

### Data extraction

We extracted data from articles in a standardised way. Weights reported in pounds (lbs) were transformed into kilograms (kg) and weeks, transformed into months. Standard errors (SE) were derived from standard deviations (SD) and the sample size. We extracted the following key data (1) Sample size, (2) Gender composition, (3) Length of follow-up, (4) Location of the study, (5) Measuring method (self-reported, third party measurement), (6) Mean weight change, (7) SD of mean weight change, (8) Mean weight change (with SD) by gender, (9) Percentage of students gaining weight, (10) Mean weight gain of weight gainers (with SD), (11) Percentage of students gaining the “Freshman 15”, (12) Statistical significance of weight gain, (13) Retention rate. Several articles did not report all of the key data. We attempted contact with all relevant authors to obtain the missing information. We assessed the quality of each study using a modified version of the Ottawa-NewCastle Scale [58]. Studies were assessed on a total score of seven on i) the representativeness of the cohort and recruitment method, ii) the outcome assessment (measurement method, time of first measurement, length of follow-up and retention rate), and iii) bias analyses conducted. Studies with scores of six and above were deemed high quality, between four and five were average quality and below four, poor quality.

### Statistical analysis

We used STATA (V.11) with the packages Metan and Metareg to analyse the data. We performed random effects meta-analysis using only studies which provided standard errors (weighted on SE and on the sample size) for the calculation of the mean weight change (baseline to follow-up). When studies reported data for multiple time points, the last time point under 8 months was used, unless stated otherwise. We used a random effects model due to the high heterogeneity. We initially dealt with missing data by contacting authors. Studies in which standard errors were not available after contacting relevant authors were imputed when possible and an imputed analysis was performed and compared to studies presenting standard errors. Imputation of SE was done using a correlation coefficient, as detailed in the Cochrane Handbook for Systematic Reviews of Interventions section 16.1.3.2(2) [59]. The SE imputation formula is based on obtaining the root-square of the SD^2^ of the baseline weight + SD^2^ of final weight – (2*SD of baseline weight* correlation factor). As detailed in the section 16.1.3.2(2), a correlation factor can be obtained for each study with SEs. These were calculated and the mean was used as the overall correlation factor. Studies with key missing data other than SE and for which we did not get an author response, were excluded from specific subgroup analyses (Table 1).

**Table 1:** Summary of included articles and selected

									Included in Analyses					
Author	Year	Country	Length (Mths)	Sample Size	Composition	Quality (on 7)	Mean WG (kg)^d^	Mean WG in Weight Gainers (kg)	1a	1b	2a	2b	2c	3
Hodge et al. [9]	1993	US	6	61	W	5	0.39	3.2			X			
Megel et al. [10]	1994	US	7	57	W	4	1.11				X			
Cooley and Toray [12]	2001	US	7	104	W	4	2.05				X			
Graham and Jones [14]	2002	US	8	49	W/M	6	−0.68	2.09			X			
Anderson et al. [6]	2003	US	3	135	W/M	4	1.3			X	X			
Levitsky et al. [16]	2004	US	2.76	60	W/M	6	1.9^b^ (2.4)		X	X				
Butler et al. [15]	2004	US	4.6	54	W	5	0.93			X				
Morrow et al. [20]	2006	US	6.7	137	W	5	1.1^b^ (2.6)		X	X				X
Hoffman et al. [18]	2006	US	7	67	W/M	5	1.3^b^ (4.0)	3.1^b^ (2.4)	X	X	X	X		X
Hajhosseini et al. [19]	2006	US	3.68	27	W/M	4	1.36^b^ (0.32)		X	X	X			
Lowe et al. [22]	2006	US	8	69	W	5	2.08				X			
Levitsky et al. (a) [21]	2006	US	2.76	15	W	5	3.1^b^ (2.0)		X	X	X			X
Levitsky et al. (b) [21]	2006	US	2.76	16	W	5	2^b^ (2.6)		X	X				X
Kasparek et al. [29]	2008	US	6	193	W/M	4	1.15	3.22		X	X			
Pliner and Saunders [30]	2008	Can.	5	72	W/M	7	1.5							
Edmonds et al. [33]	2008	Can.	6	116	W	6	2.4^c^ (3.0)		X	X				X
Delinsky and Wilson [31]	2008	US	7	149	W	3	1.53^b^ (3.4)	3.3^b^ (2.7)	X	X	X	X		X
Wengreen and Moncur [38]	2009	US	4	159	W/M	6	1.51^b^ (2.3)	4.52^b^ (1.6)	X	X	X	X		
Pullman et al. [55]	2009	Can.	7	108	M	4	3 ^c^ (4.1)		X	X				X
Mifsud et al. [34]	2009	Can.	6	29	W/M	5	0.79^c^ (2.3)		X	X				X
Provencher et al. [35]	2009	Can.	7	1323	W/M	5	1.47^c^(4.1)		X	X			X	X
Lloyd-Richardson et al. (study 2) [37]	2009	US	8	326	W/M	6	1.91^c^ (2.3)		X	X	X			
Gropper et al. [32]	2009	US	7	214	W/M	6	1.18^b^ (2.4)	2.72^b^ (2.4)	X	X	X	X	X	X
Gow et al. [39]	2010	US	1.38	40	W/M	5	0.47^b^ (1.6)		X	X				
Vella-zarb and Elgar [41]	2010	Can.	2.51	91	W/M	7	0.89^b^ (3.3)		X	X				X
Gillen and Lefkovitz [42]	2011	US	7^a^	390	W/M	5	1.18	3.3^b^ (2.6)			X	X	X	
Kapinos and Yakusheva [44]	2011	US	7	388	W/M	5	1.02^c^ (3.9)		X	X				
Webb [47]	2012	US	4	83	W	5	1.2^b^ (2.9)		X	X				X
Finlayson et al. [46]	2012	UK	3^a^	120	W/M	6	0.23^b^ (2.1)		X	X				X
Culnan et al. [51]	2013	US	1.83	54	W/M	4	0.79b (2.2)		X	X				
Deliens et al. [52]	2013	Belg.	4	101	W/M	6	0.97^c^ (2.0)		X	X	X			
Kapinos et al. [54]	2014	US	7	1935	W/M	4	0.79^b^ (3.3)		X	X				X

US = United Stated, UK = United Kingdom, W = Women, M = Men, WG = Weight Gain

^a^Studies also had a time point at 12 months but not included in analyses

^b^Standard Deviation/Standard Error presented in article

^c^Standard Deviation/Standard Error obtained by contacting authors

^d^Number of significant digit depend on the precision presented in the original articles

Subgroup analyses were performed for location, length of follow-up, gender, retention rate and weight measurement method. Meta-regressions were also performed with these covariates to investigate heterogeneity. We performed a random effects meta-analysis on the weight gained among weight gainers. Further, we did a weighted mean of the percentage of students gaining weight, percentage of students gaining 15 lb and retention rates. Heterogeneity was investigated through Galbraith plots [60] and publication bias through Funnel Plots [61]. A varying number of articles are included in each analyses. Table 1 presents the included studies by analysis.

## Results

### Studies included

**Fig. 1:**
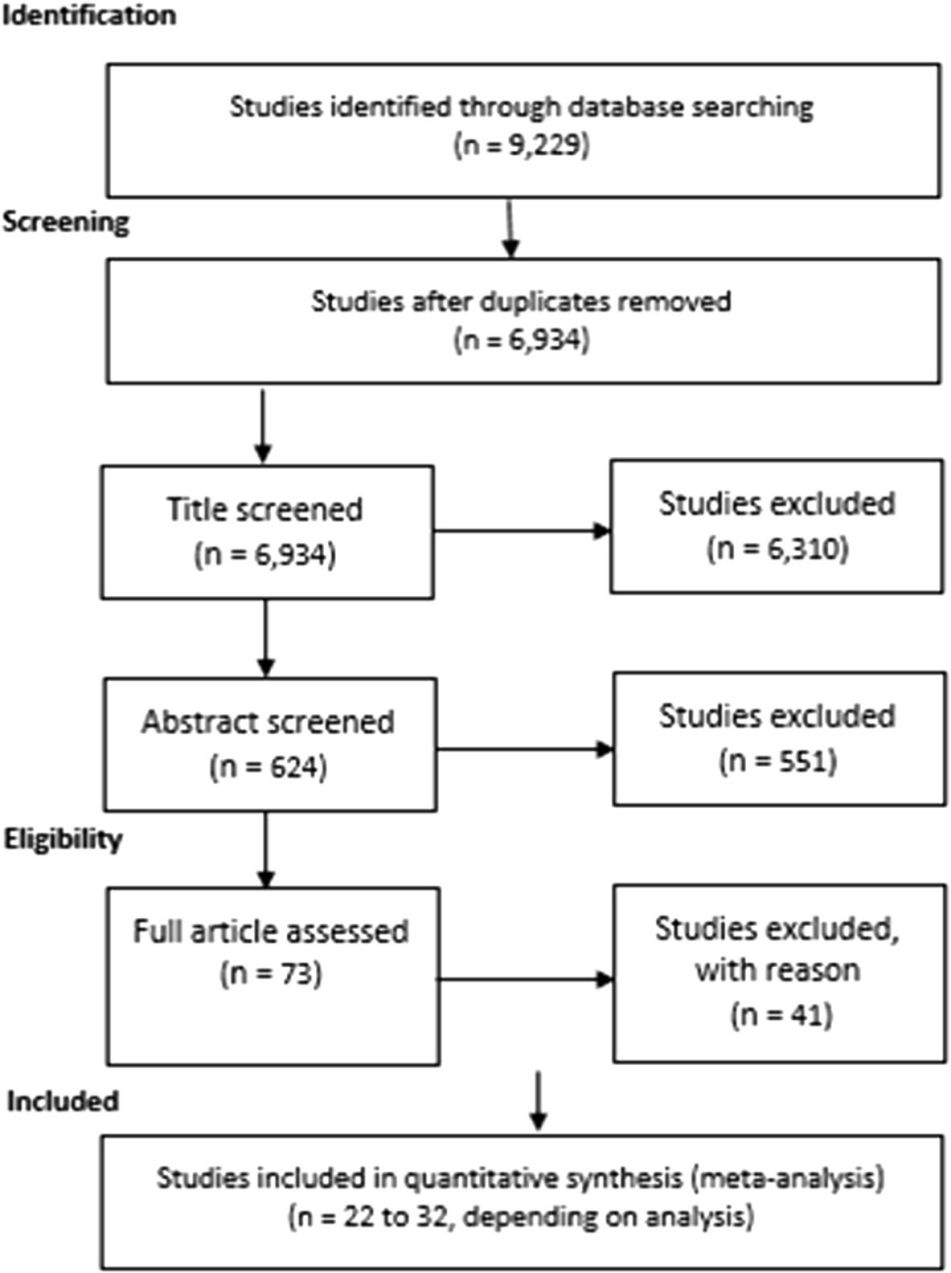
Sampling and selection of articles on weight gain in university students

We obtained 9229 records from the search performed. Of these, 32 met the inclusion criteria (Fig. 1). Ten of these studies were missing standard error for the main meta-analysis and therefore were only used in some subgroup analysis (Table 1). Studies included date from 1993 to 2014 and represented four locations: United States, Canada, United Kingdom and Belgium. All studies vary in sample size and in length of follow-up. One study reported an overall not statistically significant mean weight loss in the sample [14] whereas almost all the other studies reported an overall statistically significant mean weight gain. A summary of included studies can be found in Table 1 and a summary of all results, with results of heterogeneity tests, can be found in Table 2.

**Table 2:** Summary of random-effects meta-analysis estimates by type of analysis

	Specification	N studies	N students	Pooled estimate	Q statistics	*P*-Value of heterogeneity	I^2^
Mean weight change	Weighted on SE	22	5549	1.36 kg (CI: 1.15–1.57)	141.2	*P* < 0.001	85.1 %
Subgroup analysis	By location	15	3661	USA: 1.32 kg (1.08–1.56)	90.8	*P* < 0.001	84.6 %
		1	120	UK: 0.83 kg (0.45–1.21)	0.00	NA	NA
		5	1667	Canada: 1.71 kg (1.04–2.38)	29.7	*P* < 0.001	86.5 %
		1	101	Belgium: 0.97 kg (0.57–1.37)	0.00	NA	NA
Subgroup analysis	By body weight measurement method	4	3700	Self-report: 1.04 kg (0.63–1.44)	26.2	*P* < 0.001	88.6 %
		18	1849	Measured: 1.45 kg (1.21–1.69)	89.22	*P* < 0.001	80.9 %
Subgroup analysis	By study length	8	423	<4 mths: 1.24 kg (0.96–1.53)	44.03	*P* < 0.001	77.3 %
		14	5126	4–8 mths: 1.47 kg (1.13–1.81)	95.74	*P* < 0.001	89.6 %
Subgroup analysis	By gender	13	2876	Females: 1.34 kg (1.02–1.65)	59.59	*P* < 0.001	79.9 %
		8	1789	Males: 1.43 kg (0.90–1.97)	42.36	*P* < 0.001	83.5 %

### Mean weight change

**Fig. 2:**
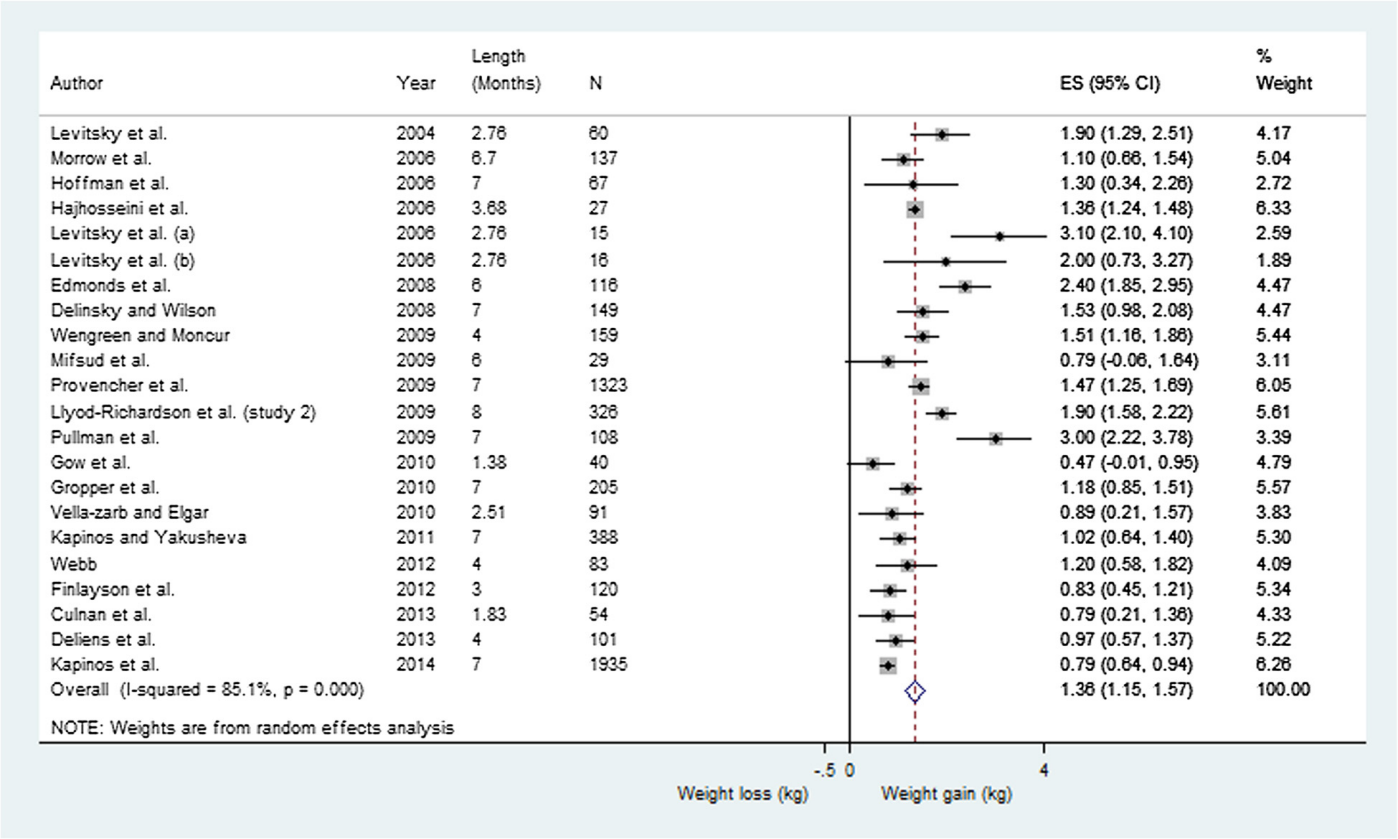
Meta-Analysis of mean (95 % CI) weight change (kg) from baseline to follow-up in first year university students; studies reporting standard errors. The overall mean weight change is 1.36 kg (CI: 1.15–1.57)

To perform a meta-analysis, the mean weight change as well as the standard error or standard deviation must be reported. Only 14 studies reported the necessary data. After contacting authors, we had sufficient data for a total of 22 studies (Table 1: Analysis 1a), accounting for a sample of 5549 students. In this analysis we observed a statistically significant weight gain of 1.36 kg (CI: 1.15–1.57, I^2^ = 85.1 %) over 6 weeks to eight months (Fig. 2). We also performed a meta-analysis weighted on sample size which yielded a mean weight gain of 1.21 kg (CI: 1.12–1.30, I^2^ = 85.2 %).

Three studies had reported enough data to allow us to impute a standard error. When these were included in the meta-analysis (Table 1, Analysis 1b), along with with 22 studies which reported standard error data, the mean weight gain was 1.35 kg (CI: 1.15–1.55). This pooled mean was not significantly different. Seven studies could not be included in the meta-analysis of mean weight change as they did not report a standard error and we were unable to obtain the missing data from authors. We conducted a Wilcoxon-Mann-Whitney test to compare the reported weight change in studies included and not included in the meta-analysis. These were not significantly different (*P* > 0.61). To avoid using imputed data and to deal with missing data, the subsequent subgroup analysis only used studies which reported standard errors (Table 1, Analysis 1a).

### Heterogeneity and bias analysis

In all meta-analyses, heterogeneity was high (above 80 %). We investigated heterogeneity through a Galbraith plot; studies were very different but no single study contributed significantly more to heterogeneity than others. The large differences in sample size and in differences in length of follow-up did contribute to heterogeneity. It is to note, that the studies had different gender composition and were conducted in different countries, which likely adds to the heterogeneity. We further performed Wilcoxon-Mann-Whitney tests to compare studies which were included and not included in the meta-analyses and sub-group analyses. In every instance, there was no statistical difference. We also analysed the potential for publication bias through a funnel plot and found a symmetric plot indicating likely low publication bias.

### Subgroup analysis by location

We further investigated weight changes according to location of study; 15 studies were conducted in the United States, five in Canada and one each in the United Kingdom and Belgium. The United Kingdom and Belgium had lower weight gain than in the United States and Canada; but inference cannot be made due to the small sample of studies. Canadian and US studies did not significantly differ in weight gain with Canada having a pooled mean of 1.71 kg (CI: 1.04–2.38, I^2^ = 86.5 %) and the United States, 1.32 kg (CI: 1.08–1.56, I^2^ = 84.6 %).

### Subgroup analysis by measurement method

In this analysis (Table 1, Analysis 1a), 18 studies measured weight change objectively while four relied on self-report. Running a sub analysis, studies using measured weight 1.45 kg (CI: 1.21–1.69, I^2^ = 80.9 %) were not significantly different than the pooled mean of those using self-reported weight, 1.04 kg (CI: 0.63–1.44, I^2^ = 88.6 %). We excluded two studies who used different weight measuring methods at baseline (self-report) and follow-up (measured) [26, 27].

### Subgroup analysis by study retention rate

We investigated the effects of the percentage of students, from the initial sample, who completed the study. The weighted retention rate was 57 %, although half of the studies had a retention rate higher than 80 %. The mean weight gain was not significant different between studies having a low retention rate (less than 40 %), medium retention rate (40–80 %) and high retention rate (above 80 %).

### Subgroup analysis by study length

The average length of the studies was five months, with a range from 6 weeks to 8 months. We investigated whether the length of studies had an impact on the reported weight change. We stratified by length of study: four months or less (representing one university term) and more than four months. Studies which had two data collection point during the year could be included a maximum of once per strata. The results showed that studies of longer length reported higher weight gain, 1.47 kg (CI: 1.13–1.81, I^2^ = 89.6 %) than shorter studies, 1.24 kg (CI: 0.96–1.53, I^2^ = 77.3 %).

As it is often reported that weight gain in 1st year university students predominately happens during the first term, we also assessed whether weight gain occurs predominately during the first term or whether it is constant over the year. Six studies had two different time points of follow-up, with reported SD. In these studies over the first four months, students gained an average of 1.24 kg (CI: 0.87–1.61, I^2^ = 81.6 %) and by the end of the academic year, they had gained on average 1.76 kg (CI: 1.32–2.21, I^2^ = 82.6 %). When we conducted a meta-regression, the length of follow-up was significant (*p* < 0.05) to predict higher weight change; the univariate R^2^ was 27.1 %.

### Weight gain in weight gainers (Analysis 2)

The overall mean weight change is affected by outliers and students losing weight. We therefore examined the percentage of students gaining weight through a weighted average of 16 studies (Table 1, Analysis 2a). A majority of students, 60.9 %, gained weight during freshman year, although studies did not have the same definition of weight gain, which is a source of heterogeneity. Some required a minimum threshold weight gain while others had none. To further investigate weight gain, we calculated weight gain in those who did gain weight only in order to get a sense of the magnitude of weight gain this subgroup. Nine studies reported the average weight gain in weight gainers but only five reported the standard deviation, required for inclusion in a meta-analysis (Table 1, Analysis 2b). The subpopulation of weight gainers gained 3.38 kg (CI: 2.84–3.92, I^2^ = 89.5 %), significantly higher than the overall pooled mean weight gain in the general first year student population. Three studies (Table 1, Analysis 2c) also reported the percentage of students gaining the “Freshman-15” 15 lb (6.8 kg). The weighted average of these studies showed that 9.3 % of 1st year students gained at least 6.8 kg.

### Analysis by gender

From the 32 studies included in this review, ten studies were conducted only with females while one study was solely conducted with males (Table 1). Twelve other studies reported weight change stratified by gender. On average, studies which had a mix sample had a higher percentage of female participants. Due to lack of reporting of standard deviations, only 14 studies could be used for a meta-analysis by gender (Table 1, Analysis 3). Females and males did not differ in their weight gain with each gaining respectively on average 1.34 kg (CI: 1.02–1.65, I^2^ = 79.9 %), and 1.43 kg (CI: 0.90–1.97, I^2^ = 83.5 %).

### Analyses by quality of study

In general, the quality was adequate with most studies ranking 5 points out of 7. Ten studies were of high quality (6–7 points), 21 of medium quality (4–5 points) and one of low quality (3 or less points). Studies of medium or low quality did not have a significantly different mean weight change than studies of high quality (*p* > 0.71). In general, studies had poor recruitment strategies and did not have a representative university sample.

## Discussion

Our meta-analysis indicated that first year university students on average gained 1.36 kg (3lbs) (CI: 1.15–1.57) over a period of 6 weeks to eight months. Importantly, it is a majority of students (60.9 %) who did gain weight during the freshman year. Within those who did gain weight, the average weight gained was 3.38 kg (7.5lbs) (CI: 2.84–3.92). We can also report from three studies that 9.3 % of first year undergraduate students gained at least 15 lb (6.8 kg). We noted through subgroup analyses that methods of data collection (self-report vs third party measurement) and retention rates did not significantly affect reported mean weight changes. We also found that males (1.43 kg) did not gain significantly more weight than females (1.34 kg). It appeared that the weight gain happened most predominately during the first term. Weight gain in the first term was 1.24 kg, only 0.5 kg less than that for the first year in total (1.76 kg). Although both figures come from pooled means, representing average overall weight gain for the samples, this could suggest that weight gain happens most predominately during the first term. No study allowed us to investigate whether the same individuals were gaining weight both during the first term and throughout the rest of the year, but these findings indicate that more than two thirds of the weight gain in first year populations happens early in their first university year. This is an important area for future research as if we were able to track weight change in individuals more confidentially, it could demonstrate whether this weight gain is non-linear and predominantly within the first 4 months as suggested from these findings, highlighting the importance of early prevention by universities. For effective health promotion efforts, further research should be conducted to evaluate individual level trends and explore this finding further.

One strength of our study is the wide systematic search performed, allowing studies from several geographical regions to be included. Further, we updated the literature with 16 recent studies to the pooled estimates. We also conducted a high quality meta-analysis using standard deviations/standard errors, leading to a more comprehensive and representative weighted analysis, all the while excluding cross-sectional studies. We approached all authors of studies with missing data in an attempt to obtain the most comprehensible dataset. We were able to receive data from 80 % of the approached authors. Importantly, we have included quality studies (quality scores above 3/7) which were prospective cohort type and which had weight measurements within the normal academic calendar. We thus excluded a few studies on these bases [9,13,17,24,36,37,48] and we were careful not to include duplicates. Our study was limited by the high degree of missing data. This yielded to several studies not being included in some of the analyses. In terms of limitation, we did not have enough data to account for baseline BMIs and ethnic make-up which limits the generality of the results. The included studies were also very different leading to high heterogeneity which limits the potential for some analyses and interpretations. Studies also had different definitions of weight gain and of ‘’significant” weight gain, as well as very different ways to present the data, making the results difficult to interpret. The previous meta-analysis had also highlighted these methodological issues [4]. Another limitation of this meta-analysis is that we rely on studies which are descriptive in nature as they have no control groups. These individual studies do not allow distinguishing the possible effect of going to university, since there is no non-university control groups.

## Conclusions

Our meta-analysis confirmed that the weight in university students increased statistically. A weight gain of 1.4 kg over two terms is meaningful and represents an increase in weight five times higher than in the general population over a year [16]. Some reviews [4, 5] have linked this weight gain to stress, alcohol drinking, unhealthy eating and poor physical activity. To our knowledge, this is the second meta-analysis to be conducted and published on this topic. The importance of this paper relies on its improvement from the previous published meta-analysis as we included 16 new studies, excluded cross-sectional studies, searched with broader terms and conducted meta-analyses weighted on standard error, as opposed to sample size. We have further conducted several novel subgroup analyses to investigate the effects of location, retention rate, measurement method and gender. Our overall pooled mean weight gain is significantly lower than the findings from the previous 2009 meta-analysis which was 1.75 kg (CI: 1.73–1.77) [4] and this can be attributable to the 16 new studies published in the field and removing all cross-sectional studies from the analysis. Indeed, the past meta-analysis included cross-sectional studies and studies which included summer months. Cross-sectional should not be included as they do not allow to show causality since the temporal relationship cannot be distinguished [62].

One important finding is that almost two thirds of students gain weight during their first year of university and they gained almost 3.5 kg (7.5lbs). Furthermore, about one in 10 students gained at least 6.8 kg (15lbs). Perhaps a shift from topic focused health promotion to a more holistic approach to health promotion including fostering healthy social and built environments could help reduce weight gain. Health promotion and health intervention seem critical in the first university year. Universities should embrace their role as potential key health promoters and shapers of student health.

Finally, this article makes a call for better quality and reporting in studies. From our systematic search, we had to exclude a number of studies due to less comprehensive data reporting or cross-sectional nature. Reported data need to be presented with standard errors/standard deviations in order to adequately interpret the findings. Further, beyond reporting overall mean weight changes, reporting the percentage of students gaining weight and the average weight gain in that sub-population should be reported. Studies on weight change should be of cohort type and be prospective. With most studies coming from North America, it would be of public health importance if more studies from other regions would investigate weight change trends in university students.
